# Next-Generation Sequencing Panel Analysis of Clinically Relevant Mutations in Circulating Cell-Free DNA from Patients with Gestational Trophoblastic Neoplasia: A Pilot Study

**DOI:** 10.1155/2020/1314967

**Published:** 2020-01-02

**Authors:** Lingxiao Luo, Ling Lin, Xiaoyan Zhang, Qingqing Cai, Hongbo Zhao, Congjian Xu, Qing Cong

**Affiliations:** ^1^Obstetrics and Gynecology Hospital of Fudan University, Shanghai 200011, China; ^2^Shanghai Key Laboratory of Female Reproductive Endocrine Related Diseases, Fudan University, Shanghai 200011, China; ^3^Institutes of Biomedical Sciences of Shanghai Medical School, Fudan University, Shanghai 200032, China

## Abstract

Gestational trophoblastic neoplasia (GTN) originates from placental tissue and exhibits the potential for invasion and metastasis. Gene alterations in GTN have not been extensively studied because of a lack of qualified tumor specimens after chemotherapy. GTN has a rapid growth rate and is highly metastatic, which makes circulating tumor DNA (ctDNA) sequencing a promising modality for gene profiling. Accordingly, in this study, we performed targeted next-generation sequencing (NGS) of 559 tumor-associated genes using circulating cell-free DNA (cfDNA) collected prior to chemotherapy from 11 patients with GTN. All sequenced genes were associated with oncogenesis, progression, and targeted therapy. The average cfDNA level was 0.43 ± 0.22 ng/*μ*L. Significant correlations were found between cfDNA concentration and maximum lesion diameter (*r* = 0.625, *p*=0.040) and time for human chorionic gonadotropin beta subunit (*β*-HCG) recovering to normal level (*r* = 0.609, *p*=0.047). There were no significant correlations between cfDNA concentrations and *β*-HCG expression level or lung metastasis. ctDNA mutations were detected in all patients, and 73 mutant genes were detected in 11 patients. *BMPR1A* (27.3%), *LRP1B* (27.3%), *ERCC4* (18.2%), *FGF14* (18.2%), *HSP90AA1* (18.2%), *KAT6A* (18.2%), *KMT2D* (18.2%), *MAP3K1* (18.2%), *RANBP2* (18.2%), and *ZNF217* (18.2%) mutations were detected as overlapping mutations. The mRNA and protein levels of bone morphogenetic protein receptor type 1A were significantly downregulated in human JAR and JEG-3 choriocarcinoma cells (*p* < 0.0001), whereas mRNA and protein levels of mitogen-activated protein kinase kinase kinase 1 were upregulated in these two cell lines (*p*=0.0128, *p*=0.0012, respectively). These genes may play important roles in GTN initiation and progression and may be candidate targets for GTN treatment. These findings suggested that cfDNA levels could provide potential assessment value in disease severity of GTN and that ctDNA sequencing was a promising approach for identifying gene mutations in GTN.

## 1. Introduction

Gestational trophoblastic neoplasia (GTN) is a cancer that originates from placental tissue and exhibits the potential for invasion and widespread metastasis. GTN includes invasive moles, choriocarcinoma, placental site trophoblastic tumors, and epithelioid trophoblastic tumor, encompassing lesions that originate in the chorionic villi and the extravillous trophoblast; these lesions exhibit different degrees of proliferation, invasion, and dissemination [1, 2]. GTN generally spreads to the lungs, liver, central nervous system, and vagina. Accordingly, detection of metastasis is essential for evaluating disease progression [3]. GTN secretes human chorionic gonadotropin beta subunit (*β*-HCG), which serves as a useful biomarker, contributing to diagnosis, monitoring of therapeutic response, early detection of relapse, and assessment of cure [4, 5].

GTN is highly vascular, and biopsy is associated with a high risk of hemorrhage; thus, histological diagnosis is not necessary for treatment. However, chemotherapy is a highly effective, first-line treatment for GTN. Most women can be cured by chemotherapy without undergoing surgery, and surgery is often only performed when chemotherapy resistance is encountered. Tumors are often destroyed by chemotherapy and cannot be used for genetic research. Therefore, gene alterations of GTN have not been extensively studied.

With the development of precision medicine, liquid biopsy has emerged as a promising, noninvasive method for analysis of circulating tumor-derived material. Liquid biopsies, including tumor circulome, cell-free DNA (cfDNA), circulating tumor DNA (ctDNA), and circulating tumor cells, represent an innovative tool in precision oncology to overcome current limitations associated with tissue biopsies [6]. GTN has a rapid growth rate and is highly metastatic, showing early hematogenous spread. Thus, ctDNA sequencing may be a promising modality for genomic profiling and a viable tool for detecting more sensitive and specific biomarkers for clinical utility. ctDNA sequencing has been reported to have potential for disease monitoring, minimal residual disease detection, and therapeutic efficacy analysis in multiple different types of cancer [7–11]. However, few studies have reported cfDNA in gestational trophoblastic tumors.

Therefore, in this study, we investigated serum levels and gene alterations of targeted cfDNA in GTN, with the goal of elucidating the potential clinical and etiological roles of cfDNA in GTN.

## 2. Materials and Methods

### 2.1. Patients and Clinical Characteristics

Patients with GTN who met the inclusion criteria were enrolled in the Obstetrics and Gynecology Hospital of Fudan University from September 2017 to September 2018. Patients were included in the study according to the following criteria: diagnosed with GTN, according to the systematization of the diagnosis and GTN staging proposed by FIGO [12]; had not undergone chemotherapy; did not have any other tumorous disease or medical comorbidities; did not have any injury history or had not received any hormone therapy within 1 year prior to enrollment; and could be followed-up. Our project was approved by the Medical Ethics Committee of the Obstetrics and Gynecology Hospital of Fudan University, and all enrolled patients signed a consent form.

### 2.2. Reagents and Instruments

Reagents included antiglyceraldehyde 3-phosphate dehydrogenase (GAPDH) antibody (cat. no. KC-5G4; Aksomics, Shanghai, China), antibone morphogenetic protein receptor type 1A (BMPR1A) antibody (cat. no. 12702-1-AP; Proteintech, IL, USA), antimitogen-activated protein kinase kinase kinase 1 (MAP3K1) antibody (cat. no. 19970-1-AP; Proteintech, IL, USA), Magnetic Serum/Plasma DNA Maxi Kit (Tiangen, Beijing, China), TRIzol reagent (Invitrogen, CA, USA), PrimeScript RT kit, and SYBR Premix Ex Taq II (Takara, Shiga, Japan). Instruments included an optical microscope, an inverted microscope and imaging system (Olympus, Tokyo, Japan), and an ABI 7900 real-time PCR system (Applied Biosystems, MA, USA).

### 2.3. Plasma Collection and Preparation

Ten milliliters of peripheral blood was collected in Cell Free DNA BCT before chemotherapy from patients with gestational trophoblastic tumors and then separated by centrifugation at 1600 ×*g* for 10 min at 4°C. The plasma layer was separated, and an additional centrifugation step at 16,000 ×*g* for 10 min was included. Plasma was stored at −80°C until analysis.

### 2.4. cfDNA Preparation

cfDNA was prepared from 3 mL plasma according to the manufacturer's instructions using a Magnetic Serum/Plasma DNA Maxi Kit (Tiangen). cfDNA was quantified using an Agilent 2100 Bioanalyzer (Agilent, CA, USA). We used a targeted NGS panel (MyGenostics, Beijing, China) in this study, which included 559 genes reported to be associated with oncogenesis, progression, and targeted therapy (Supplementary [Supplementary-material supplementary-material-1]). Whole exome sequencing of the 559 genes was performed to identify gene alterations in ctDNA from patients with GTN with a target region sequencing depth of more than 500x. DNA from paired peripheral blood mononuclear cells of the same patient was sequenced as the germline control.

### 2.5. Cell Culture

The human choriocarcinoma cell lines JEG-3 and JAR were obtained from the cell bank at the Chinese Academy of Sciences (Shanghai, China). The human trophoblast cell line HTR8/sev8 was purchased from JENNICO Biological Technology (Guangzhou, China). These cell lines were obtained within 6 months before being used in this study and were authenticated by short tandem repeat validation analysis. HTR8/sev8 cells were cultured in 1640 complete medium, and JEG-3 and JAR cells were cultured in Dulbecco's modified Eagle's medium (DMEM); both 1640 complete medium and DMEM complete medium were supplemented with 10% fetal bovine serum (Gibco, CA, USA), 100 IU/mL penicillin G, and 100 mg/mL streptomycin sulfate (Gibco).

### 2.6. Real-Time Quantitative Polymerase Chain Reaction (qPCR)

Total RNA was extracted using TRIzol reagent (Invitrogen) according to the manufacturer's instructions, followed by reverse transcription (RT) PCR using a PrimeScript RT kit (Takara) to generate cDNA. qPCR assay for multiple genes was then performed with SYBR Premix Ex Taq II (Takara) on an ABI 7900 real-time PCR system (Applied Biosystems). The *GAPDH* gene was used as a housekeeping gene. To ensure the qPCR quality, two or three primer pairs were designed for all amplification segments, but only one pair was used in the final test. Melting‐curve analyses were performed for all primers. To normalize cycle threshold (Ct) values obtained for each gene. All qPCR assays were repeated three times. The primers used for the detection of *BMPR1A*, *MAP3K1*, *HSP90AA1*, *ZNF217*, *RANBP2*, and *GAPDH* are described in Table 1.

### 2.7. Western Blotting

JEG-3, JAR, and HTR8/sev8 cells were lysed in sodium dodecyl sulfate (SDS) lysis buffer (50 mM Tris-HCl, pH 6.8, 2% SDS, 10% glycerol, 1 mM phenylmethylsulfonylfluoride, and 1 mM Na_3_VO_4_). Equal amounts of total protein were separated by SDS-polyacrylamide gel electrophoresis and then transferred to polyvinylidene fluoride membranes (Merck Millipore, MA, USA). After blocking with 5% nonfat milk for 2 h, the membranes were incubated with primary antibodies at 4°C overnight, washed, and incubated with corresponding secondary antibodies at room temperature for 60 min. The protein bands of interest were visualized by fluorography using an enhanced chemiluminescence system (Pierce Protein Biology, IL, USA). BMPR1A and MAP3K1 expression levels were quantified and normalized to the expression of the loading control (GAPDH). Means ± standard deviations of triplicate experiments were plotted by GraphPad Prism software (version 8.0).

### 2.8. Statistical Analysis

Each experiment was repeated at least three times. Statistical analyses were performed using SPSS 23.0 (IBM Corporation, NY, USA) and Prism 8.0 (GraphPad Software, CA, USA). Data are presented as the means ± standard deviations. Statistical analyses were performed with Pearson analysis and Student's *t* tests, and results with *p* values of less than 0.05 were considered significant.

## 3. Results

### 3.1. Clinical Features and cfDNA Concentrations

Eleven patients (aged 24–48 years, range: 34.1 ± 10.1 years) were enrolled in this study. Clinical characteristics are demonstrated in Table 1. Among them, 9 of 11 patients had an antecedent molar pregnancy. Pathologic results of surgical specimens identified 4 cases of choriocarcinoma. According to the International Federation of Gynecology and Obstetrics (FIGO) prognosis scoring system, eight of the eleven GTN patients were low risk (8/11, 72.7%), and three were high risk (3/11, 27.3%). Among them, patients with low‐risk GTN (FIGO risk score 0–4) was treated with the single agent actinomycin D protocols. We reduced the threshold for the use of multiple agent chemotherapy in these patients with higher FIGO risk score (5-6) because of the increased risk of resistance to single agent chemotherapy. The cure rate of treatment was 100%, and all the patients' *β*-HCG concentrations recovered to normal level within 4 months (77.7 ± 20.2 days).

**Figure 1:**
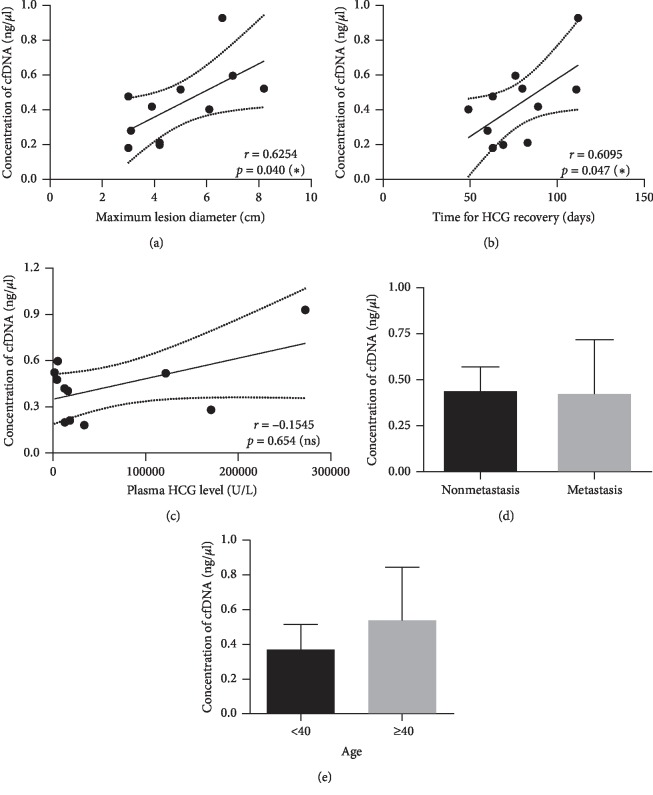
Relativity of clinical characteristics and the concentration of cfDNA. (a) Correlation of maximum lesion diameter and the concentration of cfDNA; (b) correlation of the time for *β*-HCG recovering to normal level and the concentration of cfDNA; (c) correlation of plasma HCG level and cfDNA concentration; (d) correlation of distant metastasis and cfDNA concentration (*p*=0.90); (e) correlation of age and cfDNA concentration (*p*=0.24).

We detected the cfDNA levels of all patients with GTN. The average cfDNA level was 0.43 ± 0.22 ng/*μ*L. The distribution of the maximum lesion diameter as well as the time for HCG recovery were checked using SPSS Statistics software (version 20). The Kolmogorov–Smirnov Sig. values both equaled 0.2, which was greater than 0.05; thus, the maximum lesion diameter and HCG recovery time were normally distributed. Pearson correlation analysis showed that there were correlations between the concentration of cfDNA and the maximum diameter of lesions (*r* = 0.6254, *p*=0.040; Figure 1(a)) and the time for *β*-HCG recovery to normal levels (*r* = 0.6095, *p*=0.047; Figure 1(b)). However, the distribution of *β*-HCG levels was not normal because the Kolmogorov–Smirnov Sig. value equaled 0.001 (Supplementary [Supplementary-material supplementary-material-1]). The relationship between *β*-HCG levels and cfDNA concentrations was evaluated using Spearman's correlation analysis. The correlation between plasma *β*-HCG levels and cfDNA concentrations was low (*r* = −0.1545), and the *p* value indicated a lack of significance (*p*=0.654). Moreover, Student's *t*-tests showed that there were no significant correlations between the presence of lung metastasis and the concentration of cfDNA (*p*=0.90) or patient age (*p*=0.24; Figures 1(d) & 1(e)).

### 3.2. Gene Mutation Analysis Based on Next-Generation Sequencing (NGS) Panel Analysis of Clinically Relevant Mutations in cfDNA

**Figure 2:**
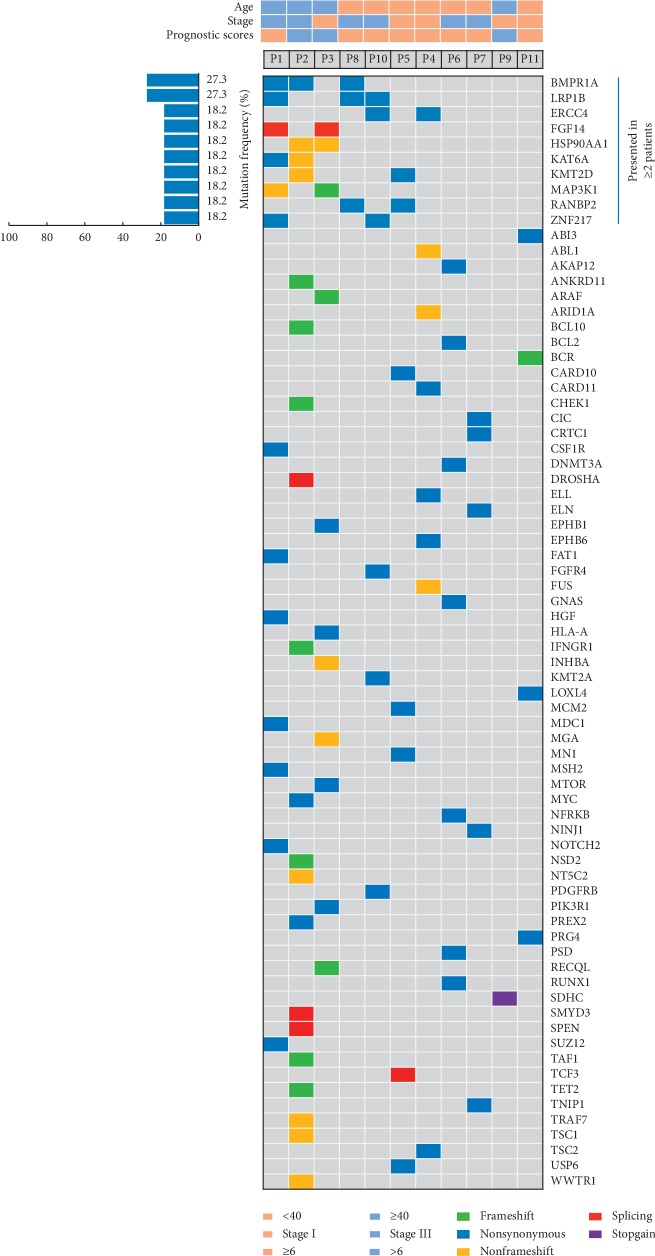
ctDNA sequencing results of GTN patients.

ctDNA mutations were detected in all patients, and 73 mutant genes were detected in 11 patients. Eleven genes, including BMPR1A (3/11, 27.3%), low-density lipoprotein receptor-related protein 1B (LRP1B; 3/11, 27.3%), excision repair cross-complementing 4 (ERCC4; 2/11, 18.2%), fibroblast growth factor 14 (FGF14; 2/11, 18.2%), heat shock protein 90 alpha family class A member 1 (HSP90AA1; 2/11, 18.2%), lysine acetyltransferase 6A (KAT6A; 2/11, 18.2%), lysine methyltransferase 2D (KMT2D; 2/11, 18.2%), MAP3K1 (2/11, 18.2%), RAN binding protein 2 (RANBP2; 2/11, 18.2%), and Zinc finger protein 217 (ZNF217; 2/11, 18.2%), showed overlapping mutations (Figure 2, Table 2).

### 3.3. Expression of Overlapping Mutations in Cell Lines

**Figure 3:**
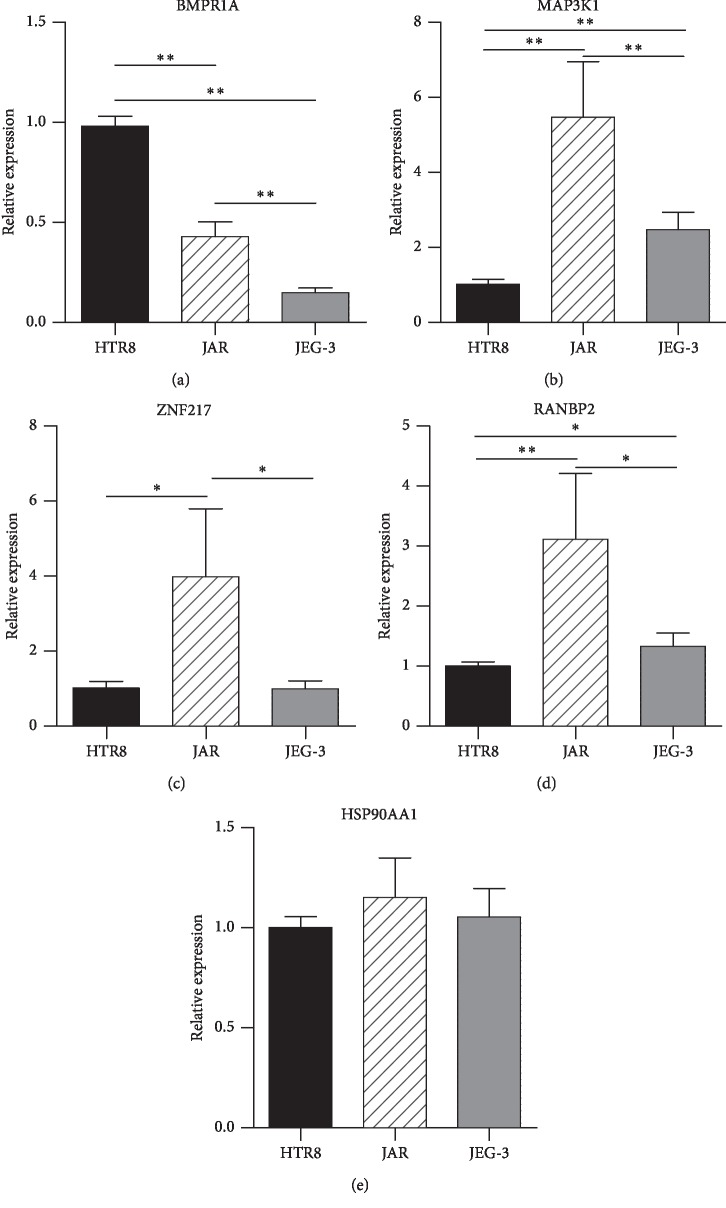
mRNA expression of mutations in cell lines (^*∗*^*p* < 0.05, ^*∗∗*^*p* < 0.001).

**Figure 4:**
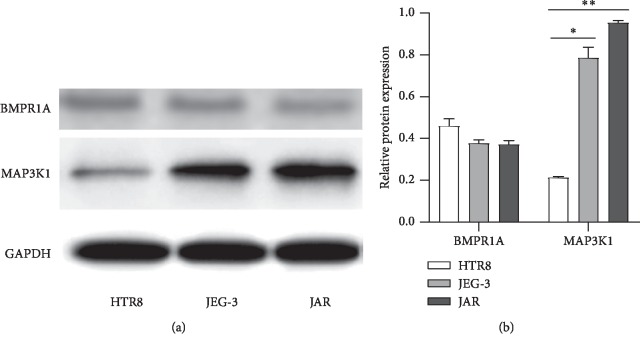
Protein expression of BMPR1A and MAP3K1 in three cell lines. (a) Western blotting gel image for BMPR1A and MAP3K1, and GAPDH was used as loading control. (b) Values of mean ± S.D. of triplicate experiments were plotted (^*∗*^*p* < 0.05, ^*∗∗*^*p* < 0.001).

The mRNA levels of BMPR1A were decreased in human JAR and JEG-3 choriocarcinoma cells (*p* < 0.0001), whereas the protein levels of BMPR1A were decreased slightly (*p* > 0.05). MAP3K1 mRNA levels were increased in JAR and JEG-3 cell lines (*p*=0.0128, *p*=0.0012, respectively), and MAP3K1 protein levels were also upregulated significantly (*p*=0.0056, *p*=0.0006, respectively). The mRNA levels of *RANBP2* and *ZNF217* were higher in JAR cell lines than in HTR8 cells (*p*=0.0008, *p*=0.0026, respectively); however, there were no significant differences between JEG-3 and HTR8 cells. There were no significant differences in *HSP90AA1* mRNA expression among these three cell lines (Figures 3 and 4).

## 4. Discussion

GTN mainly spreads by hematogenous dissemination. Therefore, in this study, we evaluated cfDNA, which provided complementary clinically relevant information for GTN. cfDNA consists of nucleic acids released from necrotic and apoptotic cells into blood circulation and is thought to be derived from both healthy and cancer cells [13]. cfDNA levels are higher in patients with cancer (0–1,000 mg/mL) than in healthy individuals (0–100 ng/mL). cfDNA analysis has been reported to have potential clinical utility in disease diagnosis and monitoring as well as early indicators of therapeutic efficacy in multiple cancer types [14–17]. However, few studies have investigated cfDNA in GTN. For example, Openshaw et al. reported that the presence of ctDNA was associated with serum hCG levels in women with GTN [18]. Additionally, in our study, we found that the maximum lesion diameter and the time for *β*-HCG recovery to normal levels were positively correlated with the levels of cfDNA in patients with GTN; thus, these factors may be used to reflect the tumor burden and evaluate the condition and prognosis of GTN, consistent with previous studies [19–21]. During tumorigenesis, apoptotic and necrotic tumor cells are engulfed by immune-related cells, such as monocytes and macrophages, thus releasing a large number of DNA fragments, which can increase the concentration of cfDNA. In addition, cfDNA levels may also increase because of the lysis of white blood cells, resulting in release of germline DNA.

We detected mutations in all pretreatment plasma samples in the 11 patients in this study, including 10 key overlapping mutant genes (*BMPR1A*, *LRP1B*, *ERCC4*, *FGF14*, *HSP90AA1*, *KAT6A*, *KMT2D*, *MAP3K1*, *RANBP2*, and *ZNF217*). These genes, particularly *MAP3K1* and *BMPR1A*, may be candidate genes in GTN initiation and progression, and could be targeted in the development of novel treatments for GTN. Sekiya et al. [22] examined the expression of nuclear factor-κB family proteins in normal placenta and choriocarcinoma cell lines; their results suggested that c-Rel may play a role in promoting the invasion of choriocarcinoma cells through phosphatidylinositol 3-kinase (PI3K)/AKT signaling. Additionally, Mello et al. [23] also revealed the involvement of phosphatase and tensin homolog (PTEN) and PI3K/Akt signaling pathways in patients with GTN. Gene ontology (GO) enrichment analysis provided support for positive regulation of several molecular functions, including regulation of DNA metabolic processes, regulation of hemopoiesis, and myeloid cell differentiation. Moreover, pathway enrichment analysis using the Kyoto Encyclopedia of Genes and Genomes (KEGG) database suggested the potential involvement of the PI3K/AKT signaling pathway, hepatocellular carcinoma, signaling pathways regulating pluripotency of stem cells, and extracellular signal-regulated kinase (ERK) 1 and ERK2 cascades.

Among the 11 patients with GTN evaluated in this study, *BMPR1A* mutations were detected in three patients (c.1001 T > C:p.L334S; 3/11, 27.3%). Interestingly, all three patients had lung metastases. Diseases associated with the protein coding gene *BMPR1A* include juvenile polyposis syndrome and polyposis syndrome [24], and this gene has been shown to be related to the mammalian target of rapamycin (mTOR) and PI3K/AKT signaling pathways [25]. Low expression of *BMPR1A* also blocks the progression and metastasis of breast cancer [26], and BMPR1A may play a role in promoting the metastasis of GTN. GTN mainly spreads by hematogenous dissemination, with the lungs being the most common distant organ for GTN metastasis. According to GO enrichment analysis, regulation of hemopoiesis could play a role in the oncogenesis of GTN. Thus, tumor cells escape from the primary site and migrate into the circulatory system, and hematopoietic cells or cytokines may infiltrate tumor sites and generate a favorable microenvironment for tumor metastasis [27, 28]. Finally, the metastatic cells proliferate and form micrometastases or macrometastases.

Overall, our study demonstrated, for the first time, that cfDNA levels could have potential value in the assessment value in disease severity of GTN and that ctDNA sequencing may be a promising approach for identifying gene alterations in GTN. In the future, additional studies with more samples are needed to further explore the value of cfDNA sequencing in patients with GTN.

## Figures and Tables

**Table 1:** Clinical characteristics of the 11 GTN patients.

Patients	Age	Antecedent pregnancy	Interval from index pregnancy (months)	Distant metastasis (number)	Maximum lesion diameter (cm)	Plasma HCG level (U/L)	Chemotherapy regimens^∗^	Surgery^#^	Pathologic results	Diagnosis	Time for *β*-HCG recovering to normal level (days)	cfDNA concentration (ng/*μ*L)
P1	48	Mole	1	Lung (6)	3.0	33740	EMA‐CO	TLH	Invasive mole	GTN III:6	63	0.181
P2	52	Mole	1	Lung (6)	6.6	>272600	EMA‐CO	TLH and BSO	Invasive mole	GTN III:9	112	0.928
P3	40	Mole	>12	No	8.2	1622	EMA‐CO	Uterine wedge resection	Choriocarcinoma	GTN I:8	80	0.522
P4	25	Mole	4	No	3.0	4209	Actinomycin D	Uterine wedge resection	Choriocarcinoma	GTN I:3	63	0.477
P5	34	Mole	3	No	3.1	170672	EMA‐CO	No	None	GTN I:5	60	0.28
P6	26	Mole	1	Lung (1)	7.0	5036	Actinomycin D	No	None	GTN III:4	76	0.596
P7	25	Mole	1	Lung (7)	4.2	12730	EMA‐CO	No	None	GTN III:5	69	0.199
P8	24	Mole	1	Lung (2)	3.9	12498	EMA‐CO	No	None	GTN III:4	89	0.419
P9	43	Abortion	3	No	5.0	121750	EMA‐CO	TLH	Choriocarcinoma	GTN I:8	111	0.517
P10	30	Abortion	2	Lung (4)	4.2	18030	EMA‐CO	D&C	Choriocarcinoma	GTN III:5	83	0.211
P11	28	Mole	4	No	6.1	15957	EMA‐CO	Uterine wedge resection	Invasive mole	GTN I:5	49	0.403

^∗^EMA‐CO: etoposide, methotrexate, actinomycin D, cyclophosphamide, vincristine. ^#^TLH: total laparoscopic hysterectomy; BSO: bilateral salphingo-oophorectomy; D&C: dilatation and curettage.

**Table 2:** Details of overlapping mutations.

No.	Gene	Transcript	Patients	Exons	Nucleotide variation	Type
1	BMPR1A	NM_004329	P1	Exon10	c.1001 T > C	SNV
			P2	Exon10	c.1001 T > C	SNV
			P8	Exon10	c.1001 T > C	SNV
2	LRP1B	NM_018557	P1	Exon39	c.6221 A > C	SNV
			P9	Exon43	c.7034 T > G	SNV
			P10	Exon39	c.6221 A > C	SNV
3	ERCC4	NM_005236	P4	Exon9	c.1853 T > A	SNV
			P10	Exon2	c.304 A > T	SNV
4	FGF14	NM_001321937	P1	Exon4	c.424-3delT	Splicing
			P3	Exon4	c.424-3delT	Splicing
5	HSP90AA1	NM_005348	P2	Exon5	c.836_838delAGA	Deletion
			P3	Exon5	c.836_838delAGA	Deletion
6	KAT6A	NM_006766	P1	Exon2	c.282 A > T	SNV
		NM_006766	P2	Exon16	c.3218_3219insGGA	Insertion
7	KMT2D	NM_003482	P2	Exon13	c.4059_4061delGGA	Deletion
		NM_003482	P5	Exon10	c.1301 T > G	SNV
8	MAP3K1	NM_005921	P1	Exon14	c.2917_2918insCTC	Insertion
			P3	Exon20	c.4487delG	Deletion
9	RANBP2	NM_006267	P5	Exon16	c.2275 G > A	SNV
			P8	Exon20	c.6680 A > G	SNV
10	ZNF217	NM_006526	P1	Exon3	c.1645 G > A	SNV
			P10	Exon3	c.1645 G > A	SNV

## Data Availability

The data used to support the findings of this study are available from the corresponding author upon request.

## Supplementary Materials

Supplementary Materials Supplementary Table 1. A targeted NGS panel including 559 genes. Supplementary Table 2. Normal distribution test for plasma HCG level.
