# Supplementary material for: Next-Generation Sequencing Panel Analysis of Clinically Relevant Mutations in Circulating Cell-Free DNA from Patients with Gestational Trophoblastic Neoplasia: A Pilot Study
Source: Biomed Res Int. 2020 Jan 2;2020:1314967. doi: 10.1155/2020/1314967 (PMC6970497; doi:10.1155/2020/1314967)
Supplement: Supplementary Materials — Supplementary Table 1. A targeted NGS panel including 559 genes. Supplementary Table 2. Normal distribution test for plasma HCG level. [file 1314967.f1.pdf]

Supplementary Table 1. A targeted NGS panel including 559 genes.

ABL1	CDK6	FAM46C	ID3	MSI1	PPP2R1A	SLX4
ABL2	CDK8	FANCA	IDH1	MSI2	PPP2R2A	SMAD2
ABRAXAS1	CDKN1A	FANCC	IDH2	MST1	PPP4R2	SMAD3
ACVR1	CDKN1B	FANCD2	IFNGR1	MST1R	PPP6C	SMAD4
ACVR1B	CDKN2A	FANCE	IGF1	MTAP	PRDM1	SMARCA4
ADGRA2	CDKN2B	FANCF	IGF1R	MTOR	PRDM14	SMARCB1
AGO2	CDKN2C	FANCG	IGF2	MUTYH	PREX2	SMARCD1
AKT1	CEBPA	FANCL	IKBKE	MYB	PRKAR1A	SMO
AKT2	CENPA	FAS	IKZF1	MYC	PRKCI	SMYD3
AKT3	CHD2	FAT1	IL10	MYCL	PRKD1	SNCAIP
ALK	CHD4	FBXW7	IL7R	MYCN	PRKDC	SOCS1
ALOX12B	CHEK1	FGF10	INHA	MYD88	PRKN	SOS1
AMER1	CHEK2	FGF12	INHBA	MYOD1	PRSS8	SOX10
ANKRD11	CIC	FGF14	INPP4A	NBN	PTCH1	SOX17
APC	CREBBP	FGF19	INPP4B	NCOA3	PTEN	SOX2
AR	CRKL	FGF23	INPPL1	NCOR1	PTP4A1	SOX9
ARAF	CRLF2	FGF3	INSR	NEGR1	PTPN11	SPEN
ARFRP1	CSDE1	FGF4	IRF2	NF1	PTPRD	SPOP
ARID1A	CSF1R	FGF6	IRF4	NF2	PTPRO	SPRED1
ARID1B	CSF3R	FGFR1	IRS1	NFE2L2	PTPRS	SPTA1
ARID2	CTCF	FGFR2	IRS2	NFKBIA	PTPRT	SRC
ARID5B	CTLA4	FGFR3	JAK1	NKX2-1	QKI	SRSF2
ASXL1	CTNNA1	FGFR4	JAK2	NKX3-1	RAB35	STAG2
ASXL2	CTNNB1	FH	JAK3	NOTCH1	RAC1	STAT3
ATM	CUL3	FLCN	JUN	NOTCH2	RAC2	STAT4
ATR	CUL4A	FLT1	KAT6A	NOTCH3	RAD21	STAT5A
ATRX	CXCR4	FLT3	KDM5A	NOTCH4	RAD50	STAT5B
AURKA	CYLD	FLT4	KDM5C	NPM1	RAD51	STK11
AURKB	CYP17A1	FOXA1	KDM6A	NRAS	RAD51B	STK19

AXIN1	CYSLTR2	FOXL2	KDR	NSD1	RAD51C	STK40
AXIN2	DAXX	FOXO1	KEAP1	NSD2	RAD51D	SUFU
AXL	DCUN1D1	FOXP1	KEL	NSD3	RAD52	SUZ12
B2M	DDR1	FRS2	KIT	NT5C2	RAD54L	SYK
BABAM1	DDR2	FUBP1	KLF4	NTHL1	RAF1	TAF1
BAP1	DICER1	FYN	KLHL6	NTRK1	RANBP2	TAP1
BARD1	DIS3	GABRA6	KMT2A	NTRK2	RARA	TAP2
BBC3	DNAJB1	GATA1	KMT2B	NTRK3	RASA1	TBX3
BCL10	DNMT1	GATA2	KMT2C	NUF2	RB1	TCF3
BCL2	DNMT3A	GATA3	KMT2D	NUP93	RBM10	TCF7L2
BCL2L1	DNMT3B	GATA4	KMT5A	NUTM1	RECQL	TEK
BCL2L11	DOT1L	GATA6	KNSTRN	P2RY8	RECQL4	TERC
BCL2L2	DROSHA	GID4	KRAS	PAK1	REL	TERT
BCL6	DUSP4	GLI1	LATS1	PAK3	RET	TET1
BCOR	E2F3	GNA11	LATS2	PAK5	RFWD2	TET2
BCORL1	EED	GNA13	LMO1	PALB2	RHEB	TGFBR1
BCR	EGFL7	GNAQ	LRP1B	PARP1	RHOA	TGFBR2
BIRC3	EGFR	GNAS	LTK	PARP2	RICTOR	TIPARP
BLM	EIF1AX	GPS2	LYN	PARP3	RIT1	TMEM127
BMPR1A	EIF4A2	GREM1	LZTR1	PAX5	RNF43	TMPRSS2
BRAF	EIF4E	GRIN2A	MAF	PAX8	ROS1	TNFAIP3
BRCA1	ELF3	GRM3	MAGI2	PBRM1	RPS6KA4	TNFRSF14
BRCA2	ELOC	GSK3B	MALT1	PDCD1	RPS6KB2	TOP1
BRD4	EMSY	H3F3A	MAP2K1	PDCD1LG2	RPTOR	TOP2A
BRIP1	EP300	H3F3B	MAP2K2	PDGFRA	RRAGC	TP53
BTG1	EPAS1	H3F3C	MAP2K4	PDGFRB	RRAS	TP53BP1
BTG2	EPCAM	HDAC1	MAP3K1	PDK1	RRAS2	TP63
BTK	EPHA3	HGF	MAP3K13	PDPK1	RSPO2	TRAF2
CALR	EPHA5	HIST1H1C	MAP3K14	PGR	RTEL1	TRAF7
CARD11	EPHA7	HIST1H2BD	MAPK1	PHOX2B	RUNX1	TSC1

CARM1	EPHB1	HIST1H3A	MAPK3	PIK3C2B	RUNX1T1	TSC2
CASP8	EPHB4	HIST1H3B	MAPKAP1	PIK3C2G	RXRA	TSHR
CBFB	ERBB2	HIST1H3C	MAX	PIK3C3	RYBP	TYRO3
CBL	ERBB3	HIST1H3D	MCL1	PIK3CA	SDC4	U2AF1
CCND1	ERBB4	HIST1H3E	MDC1	PIK3CB	SDHA	UPF1
CCND2	ERCC2	HIST1H3F	MDM2	PIK3CD	SDHAF2	VEGFA
CCND3	ERCC3	HIST1H3G	MDM4	PIK3CG	SDHB	VHL
CCNE1	ERCC4	HIST1H3H	MED12	PIK3R1	SDHC	VTCN1
CCNQ	ERCC5	HIST1H3I	MEF2B	PIK3R2	SDHD	WISP3
CD22	ERF	HIST1H3J	MEN1	PIK3R3	SESN1	WT1
CD274	ERG	HIST2H3C	MERTK	PIM1	SESN2	WWTR1
CD276	ERRFI1	HIST2H3D	MET	PLCG2	SESN3	XIAP
CD70	ESR1	HIST3H3	MGA	PLK2	SETD2	XPO1
CD74	ETV1	HLA-A	MITF	PMAIP1	SF3B1	XRCC2
CD79A	ETV4	HLA-B	MKNK1	PMS1	SGK1	YAP1
CD79B	ETV5	HNF1A	MLH1	PMS2	SH2B3	YES1
CDC42	ETV6	HOXB13	MPL	PNRC1	SH2D1A	ZBTB2
CDC73	EWSR1	HRAS	MRE11	POLD1	SHOC2	ZFHX3
CDH1	EZH1	HSD3B1	MSH2	POLE	SHQ1	ZNF217
CDK12	EZH2	HSP90AA1	MSH3	PPARG	SLC34A2	ZNF703
CDK4	EZR	ICOSLG	MSH6	PPM1D	SLIT2	

Supplementary Table 2. Normal distribution test for plasma HCG level.

	Kolmogorov-Smirnov[a]			Shapiro-Wilk		
	statistic	df	Sig.	statistic	df	Sig.
VAR00001	.346	11	.001	.703	11	.001

a. Lilliefors correction to significance level
